# Reserves quantity and economic stability: the central bank of Ghana's position and practices

**DOI:** 10.1016/j.heliyon.2019.e02856

**Published:** 2019-12-12

**Authors:** Abdul-Rashid Abdul-Rahaman, Hongxing Yao

**Affiliations:** School of Finance and Economics, Jiangsu University, 301 Xuefu Road, Zhenjiang, PR China

**Keywords:** Economics, Vector error correction model, Balance of payment variables, Central bank reserves, Central banks, Ghana

## Abstract

Discussions on Reserve Adequacy Assessment on a country-specific basis have been scant in the literature despite its significance. This situation has led to calls for the discussions on reserves holdings and adequacy levels to be more rooted in the country's Article IV consultation report.

In this study, we examined if reserves build-up by the central bank of Ghana meets the specific metrics of theoretical reserve models such as the reserves to M2 growth metrics and the Greenspan-Guidotti rule metrics. We also determined whether the reserves build-ups correctly adjust to economic and policy variables and whether these adjustments are consistent with the predictions of empirical theories and findings. We again examined whether exchange rate depreciations contribute to positive reserves accumulation by the central bank of Ghana.

Using Vector Error Correction Model, this research has rejected the short-run matching of the reserves stock of the central bank of Ghana to the theoretical standard metrics of 20% of broad money and 100% of short-term debts. These results have implications on the future credit ratings of the country and could also make future borrowing more expensive. Also, the impulse response functions showed a mixed result of the adjustments in reserves build-up to the predictions of empirical theories and findings. Additionally, the analysis proved the long-run test of restriction of exchange rate depreciation on positive reserves build-up of the central bank. However, the short-run adjustments of the exchange rates on reserves rejected this relationship. Furthermore, the decomposition analyses showed that short-run variations in reserves are explained by external forces, whereas the long-run variations are explained through the financial sector and partly by external forces. Therefore, an econometric specification of reserves beyond three months must specify in the model both the financial sector and external sector variables.

## 1 Introduction

The amount and composition of foreign exchange reserves have been at the center stage of central banks' management since decades (Roger, 1993; Obstfeld et al., 2010). However, there is still little agreement on how to assess and estimate the reserves holdings or adequacy levels even though this is an important aspect of a country's external stability assessment (International Monetary Fund, 2015). The changing patterns of international trade, institutional changes in the economy, and structural shifts in production are all factors which influence the quantity and composition of reserves held by a central bank (Central Bank of Nigeria, 2018). The need for central banks to always assess and keep at least the minimum amount of reserves is fundamental to their duties as regulators, and a failure in keeping to the minimum reserves required could distort the smoothness of a country's trade when shocks hit the economy. This can also cause frequent instability in a country's currency as well as in other major macroeconomic variables. In today's open market and highly liberalized global financial system, most central banks see the reserves as an alternative for capital control measures (Steiner, 2013), and their significance as a policy tool, according to Roger (1993), can only be minimized by a well-developed domestic financial market.

Ghana, like most developing countries, is faced with under-developed and weak financial systems that threaten the successful operation of the credit-view channel of monetary policy (see Banking sector Article IV Consultation Report, 2017, Quartey and Afful-Mensah, 2014 and IMF policy Article IV Consultation Report, 2017; Amissah-Arthur, 2010). This situation, coupled with the apparent inability of monetary authorities to anchor the exchange rates and to create an environment of stability, has made reserves in Ghana the most viable tool for the central bank's grist of the economy (Roger, 1993).

The need to research on Ghana's reserve adequacy assessment is fundamental to the stability of the economy, and will also serve to highlight the sovereign risks of the country to the growing segment of investors since the discovery of oil and its commercialization in 2009. The vulnerability of the country to capital flights and trade imbalances is apparent by the large percentage of investment held in the country by foreigners. For example, the IMF article IV Article IV Consultation Report (2013) showed that foreign creditors own one-third of domestic government debts. This is notwithstanding the fact that the country's imports far exceed its export potential. A confluence of adverse developments could therefore severely weaken the balance of payments position of the country.

In this study, we have examined the adequacy of Ghana's reserves holdings in line with the theoretical metrics such as the Import metrics, Reserves to M2 growth metrics and the Greenspan-Guidotti rule metrics. We have also tested whether the depreciation of the cedi is a planned strategy for the building of reserves by the central bank. This objective is against the backdrop in Pina (2015) and Aizenman and Sun (2012) that reserve accumulation by a central bank could be through currency depreciation. Lastly, this research has suggested model specifications for the forecasting of reserves within specify time horizons through decomposition analysis.

The literature on central banks’ foreign exchange reserves holdings and adequacy levels have been vast and broadly classified into two groups. The first group of researchers, such as Jeanne and Ranciere (2011), Victor and Vladimir (2006), Garcia and Soto, 2004, Bird and Rajan, 2003, Calvo and Reinhart (2002), and Rodrik and Velasco (1999) has matched the reserve adequacy levels with developments in economic variables such as the import levels, broad money (M2), and short-term debts levels. The second group which includes Tule et al. (2015), Ben-Bassat et al. (1992), Heller (1966), Hamada and Ueda (1977), and Frenkel and Jovanovic (1981), are concerned with the cost-and-benefit associated with the holding of these reserves in the central banks as pre-requisites for the determination of the reserve adequacy levels. Also, whiles a lot of researchers and IMF policy paper discussions have encouraged country-specific circumstances in determining the level of reserves; other researchers have come out with global or general standards or metrics. This is especially true for the first group of researchers. For example, Triffin (1960) suggests a general reserve holding of 30 percent of broad money or four (4) months of imports. In another development, Wijnholds and Kapteyn (2001) suggest a general threshold of around 10 to 20 percent of broad money, and Greenspan (1999), and Bussière et al. (2015) state a threshold equal to the amount of short-term debts.

There are many other generalized thresholds in the literature on reserve adequacy. However, the majority of these theoretical metrics adopt highly restrictive assumptions that limit the number and type of countries to which such analyses are likely to be reasonably applied. Roger (1993) mentions that most of the theoretical generalization is likely to be best apply to countries that are price takers in the world markets, have fairly unrestrictive current account flow and maintains a reasonably fixed exchange rate regime. The IMF policy paper and Board discussions on reserve adequacy, therefore, have encouraged analyses based on country-specific circumstances by outlining a framework as a guide for the discussions (ARA (2013) cited by International Monetary Fund, 2015).

This paper has been designed to examine if reserves build-up by the central bank of Ghana meets the specific metrics of theoretical reserve models such as the reserves to M2 growth metrics and the Greenspan-Guidotti rule metrics. We also determined whether the reserves build-ups correctly adjust to economic and policy variables and whether these adjustments are consistent with the predictions of empirical theories and findings. We again examined whether exchange rate depreciations contribute to positive reserves accumulation by the central bank of Ghana.

This research has concluded that the short-run matching of the reserves stock of the central bank of Ghana to the theoretical standard metrics of 20% of broad money and 100% of short-term debts have not been met. Also, the research found a mixed result in the impulse response functions, evident by the adjustments in reserves build-up, to the predictions of empirical theories and findings. Additionally, the test results accepted the long-run test of restriction of exchange rate depreciation on positive reserves build-up of the central bank and rejected the short-run adjustments of the exchange rates on reserves. Furthermore, the decomposition analyses in the research showed that short-run variations in reserves are explained by external forces, whereas the long-run explained variance is through the financial sector and partly by external forces. The research, therefore suggests an econometric specification of reserves beyond three months to specify in the modeling both the financial sector and external sector variables.

This paper was organized into four sections. Section one (1) introduces the research topic and state the significance of the research. Section two (2) is the literature review which contains literature on foreign exchange reserves and the profiling of Ghana's financial system as well as the objectives of the central bank of Ghana. Section three (3) presents the methodology of the research and section four (4) concludes the research findings.

## 2 Literature review

As openness, financial integration, and occasional intervention in the foreign exchange market has become an overriding objective for most economies in the world, the need to keep and manage enough reserves to insure against the risk of fluctuations has become an objective for most central banks globally (Obstfeld et al., 2010). According to Roger (1993), central banks will need to adapt their reserve stock to cater for the increasing currency convertibility and changes in exchange rate arrangements. However, as discussions in the Assessment of Reserve Adequacy (ARA) (2013) noted, reserves could play different roles in advanced, emerging, and low-income economies. The amount, composition and management of foreign exchange reserves have been, and still is, an essential topic in macro-finance and economics. Many researchers have suggested various motives for the holding of these reserves, but in the survey by ARA (2013), about three-quarters of the respondent countries’ authorities viewed precautionary liquidity needs as the critical reason to hold reserves (ARA, 2013 cited by International Monetary Fund, 2015).

Several factors have influenced the evolution of foreign exchange reserves such as the changing patterns of international trade, institutional changes in the economy, and structural shifts in production (Central Bank of Nigeria, 2018). The fear of capital mobility by some central banks may also influence the amount and composition of foreign exchange, and most central banks may use this approach as a substitute for capital control measures (Steiner, 2013). However, these factors seem to have a mixed influence on the economies, partly because of the motive of a country for holding reserves, and also partly due to the level of financial sector development and integration. For example, Roger (1993) and Pina (2015) noted that, in an economy where capital markets are well developed, both the private and public sector could easily have access to foreign exchange or contingent borrowings in times of crisis to finance international transactions. This may reduce the central bank's holding of reserves. They further noted that countries with well-developed domestic financial markets would have less need for reserves using reserves for intervention purposes, because of the ability of the markets to self-correct itself in the medium to long term.

**Figure 1:**
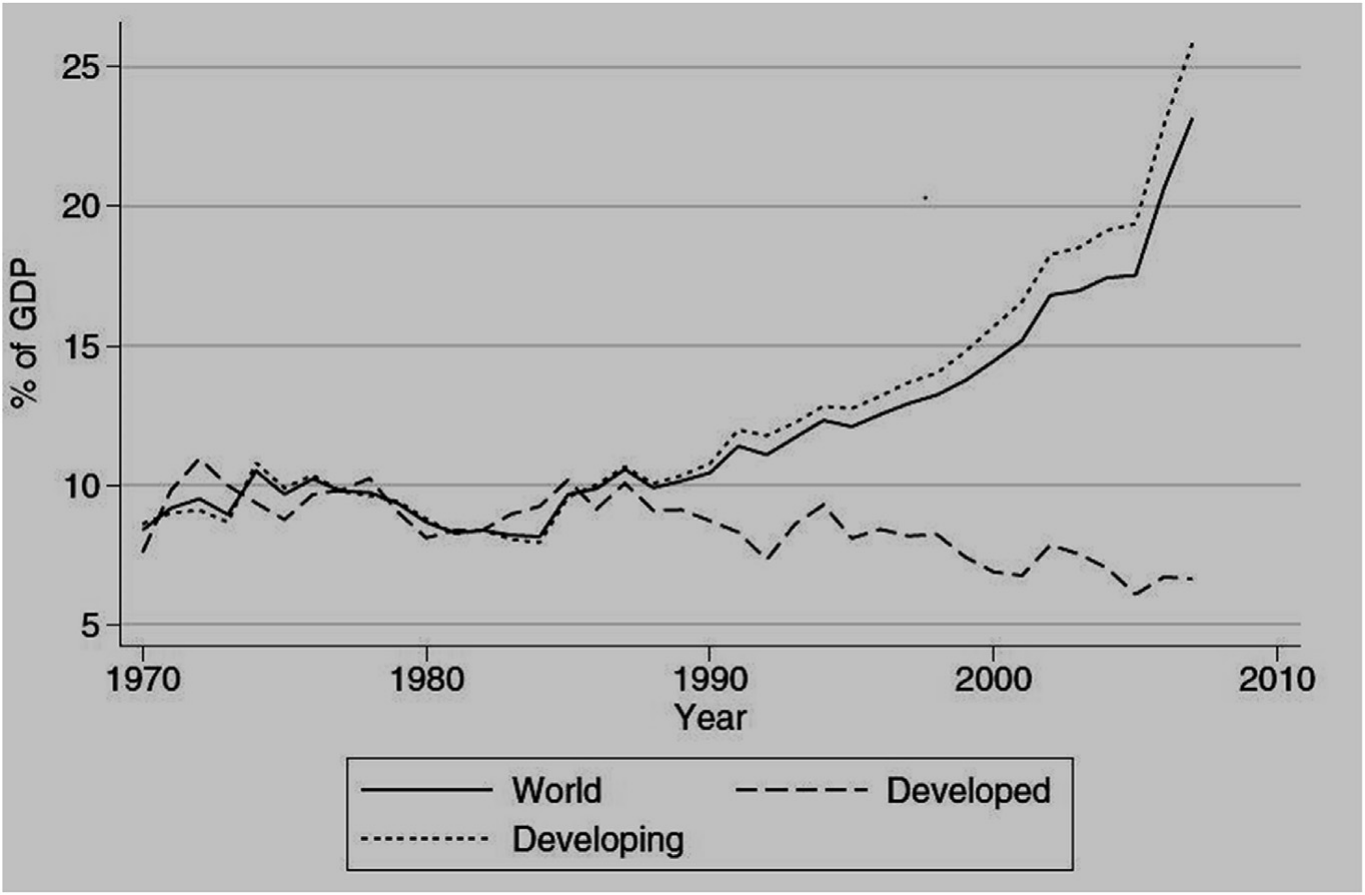
Reserve Growth for developed and developing economies. Source: Pina (2015). Permission for reuse of Figure Granted.

Furthermore, the production structure of an economy such as a commodity-intensive economy also has its toll on a country's reserve (ARA, 2013). These situations make the holding of reserves differ for different countries with different circumstances and motives. Pina (2015) working on international reserve growth for 24 developed economies and 154 developing economies between 1970-2009, observed that whiles the reserves of developed nations are shrinking, that of their developing counterparts are increasing. Figure 1 presents these differences in reserves.

**Figure 2:**
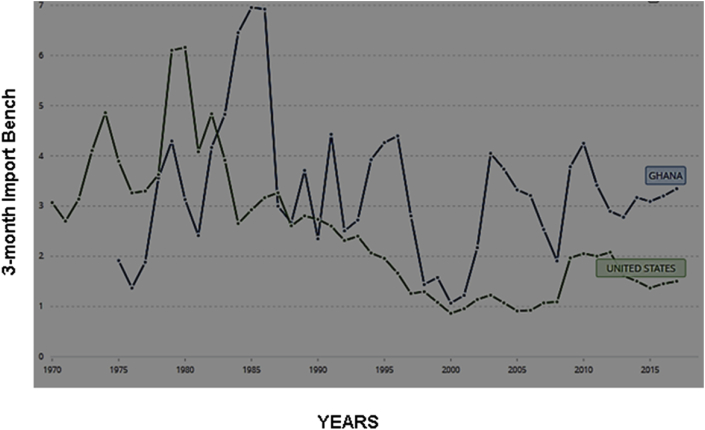
Difference in Reserve holdings of Ghana and the US (Total reserves in months of imports). Souce: World Bank data (1970–2017). Permission for reuse of Figure Granted.

We also compared the reserve holdings of Ghana (a developing country), with the reserve holdings of the United States (a developed nation) from 1970-2017 in Figure 2. These two scenarios defy the conventional logic for holding reserves for precautionary purposes. That is, developed nations are more open to both the goods and the international capital markets and coupled with the enormous volumes of international transactions, are more vulnerable to exposure than the developing countries, and should have kept more reserves by any standard; but that has proven not to be the case. The evidence provided in IMF policy paper and discussions (2015) has done its best in explaining this phenomenon. The situation implies upward movement of capital; that is, a movement of scarce resources from the developing countries to the developed nations. This contributes to the global imbalances problem currently in international finance and economics.

### 2.1 The financial sector reforms in Ghana

**Figure 3:**
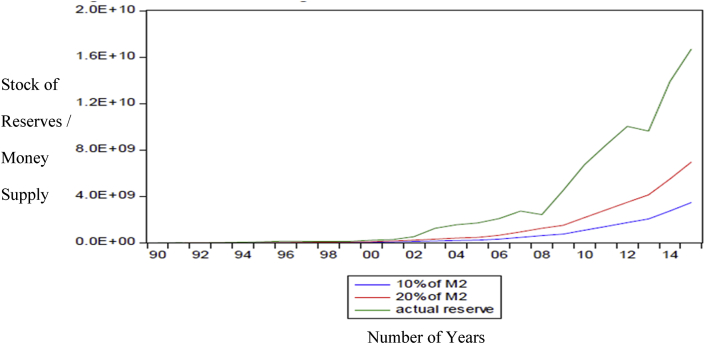
Relationship between reserves and money supply in Ghana. Source: Yao and Abdul Rashid (2018). Permission for reused of Figure Granted.

Ghana's indirect approach to monetary policy started only in 1992 at the start of the financial sector reforms (Quartey and Afful-Mensah, 2014; Bawumia et al., 2008). The banking and financial sector was growing, and there was a tremendous expansion in the money supply due to the realization of alternative ways of conducting business or exchanging goods either than the traditional use of M1. The instability in money demand function resulting from the innovations and increasing technology in the financial sector led to the collapse of the monetary aggregate policy (Driscoll et al., 1984). This made several developing countries, through IMF and World Bank directives, to implement an Economic Reform Program (ERP) which targeted mostly the banking and financial sector. According to Quartey and Afful-Mensah (2014), Ghana in the indirect control mechanism in 1992, moved from a monetary target to an inflation target after several substitutes for money emerged, and the monetary target started to fail. As part of the reform programs, Central Banks were to adapt their reserves to counter the adverse effects of the increased money supply. Researching on the relationship between reserves and money supply in an economy, Lane and Burke (2001) found a positive correlation between the two. Yao and Abdul Rashid (2018), and Obstfeld et al. (2010) also showed the positive relationship between foreign exchange reserves and money supply. Figure 3 shows a plot of reserves growth and money supply in Ghana.

Furthermore, the undervalued exchange rates are linked continuously to the considerable stock of international reserves. In the model, the central bank accumulates international reserves by resorting to inflation. This reduces the value of the domestic currency relative to foreign currencies. This was the view supported by Aizenman and Sun (2012) and Dominguez et al. (2012).

**Figure 4:**
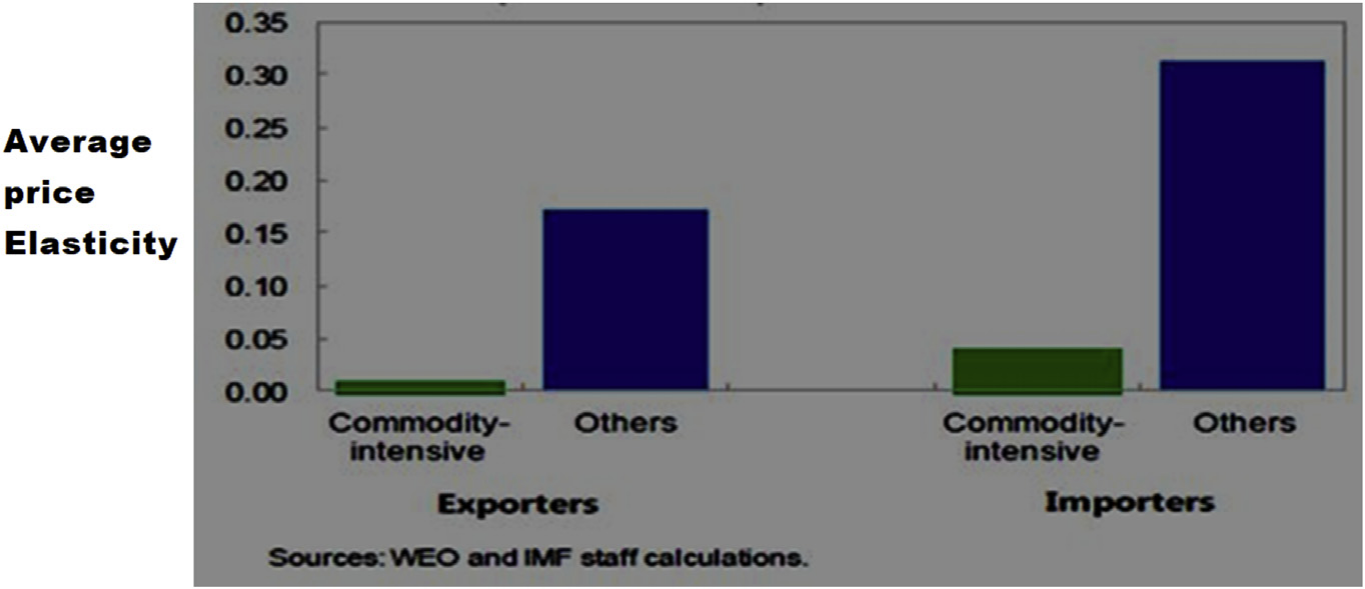
Average price elasticity of commodity-intensive economies, 1985–2013 (absolute values). Permission for reuse of Figure Granted.

Additionally, some of the developing economies are generally commodity-intensive economies, and this makes their terms of trade more volatile to shock. Ghana has suffered from a long narrow revenue base because of over-reliance on primary commodities traded in its raw state without further processing (Lolol, 2014). The ARA (2013 cited by International Monetary Fund, 2015) report mentions that the need for countries’ with deepening financial markets should analyze their reserves in two stages. Firstly, on the general metric, and second by the country-specific operational issues. This process is an adjustment to the general metric to provide for the additional risk of being a commodity-intensive economy. The recommendation in International Monetary Fund (2015) is that the reserve holdings of a country should be analyzed in details in its article iv consultation report, by spelling out the general metric and any specific risk to the economy. The additional risks for being a commodity-intensive economy could be because exports and imports are relatively priced inelastic and highly volatile, and therefore higher reserves or other alternatives may be required to smooth the adjustment process. This is because; most commodity-intensive countries will depend on the prices of their exports on the commodity market to build-up their international reserves, and strong terms of trade could help in bringing in foreign assets for this purpose. Figure 4 shows the price elasticity of commodity-intensive economies.

### 2.2 General metrics for measuring reserves

There are several proposed metrics for measuring reserve adequacy of central banks. Whiles most of the metrics depend on economic fundamentals; others depend on optimization principles. Roger (1993) mentions that each metric is particularly suited to solving a specific risk problem, and therefore the selection of a metric must be carefully carried out. Reserves can bring considerable benefits to the country that holds them, but also entails costs as they generally yield lower returns than alternative uses International Monetary Fund (2015). Ghana's official reserves are lower as compared to its sub-Saharan peer group and rule-of-thumb benchmarks (IMF Article IV Consultation Report, 2013). ARA (2013) mentions that the metrics for assessing reserve adequacy is only relevant in less matured economies; described as countries with deepening financial market. However, for developed and matured economies, the need for holding reserves to mitigate against external risk is seen as inconsequential and can be waived (ARA, 2013 cited by International Monetary Fund (2015); Roger, 1993). The reason cited for this is the ease in foreign exchange access for transactional and non-transactional motives in those countries. Also, countries whose currencies are reserve currencies are said to have little exposure to risk.

The commonly known metrics in literature are import cover, the ratio of reserves to short-term debts, the ratio of reserves to broad money and combination metrics. Each metric has its merits and may be suitable for a particular objective of the government. There are also considerations for selecting a metric or a combination of metrics. For instance, countries with matured financial and banking sector and a liberalized capital account may adopt the ratio of reserves to M2 as a preferred metric. Also, countries with rising short-term external debts may adopt the Greenspan-Guidotti rule of 100 percent cover of short-term debts, and for countries with extensive capital control measures, the import cover is suitable. There are however countries that need two or more metrics to protect different risks in the economy. For example, the expanded Greenspan-Guidotti rule consists of providing cover for short-term debts plus the current account deficit. This is intended to brace the economy for the full potential 12-month financing need. Also, Wijnholds and Kapteyn (2001) used short-term debts and M2 to model debt repayment and capital outflows as motivation for holding reserves.

The IMF Article IV Consultation Report (2017) has bemoaned the lack of adequacy and urgency of Ghana's International reserves stock in mitigating against external risks and exposures. The hypotheses in section 3.1 were therefore formulated to test whether Ghana's reserves stock are in line with any empirical benchmarks, and also to examine the strategy of reserves accumulation by the central bank of Ghana.

### 2.3 Ghana's external exposure and the benefits for holding reserves

The benefits of holding reserves and the motives for keeping them are linked together. These motives are different for each country and each circumstance. Among the reasons cited for holding reserves by International Monetary Fund (2015) are to assure confidence in the national currency; counter disorderly market conditions; support the conduct of monetary policies; build assets for intergenerational purposes or; influence the exchange rate. There are also costs associated with the holding of these reserves. Therefore, the economic decision for any additional reserve kept should be a comparison of the marginal cost and benefits of keeping the reserves.

A country's need for reserves is mostly associated with its degree of openness or exposure to external fluctuations, and Ghana's economy is hugely vulnerable in this regard. The fear and risk of capital flight are becoming increasingly worrying because of the large inflows of capital and goods into the economy. Ghana is seeking to raise $10 billion in foreign direct investment (FDI) alone compared to the $4.91 billion raised in 2017. Ghana is also targeting a rise in FDI between $20 billion and $40 billion within the next six to ten years (Daily Guide Africa, 2016). Though Herzer et al. (2007) do not find any association between economic growth and FDI, the threat inward-FDIs pose to the economic stability of a country is not in dispute. The injection of foreign currency into the economy puts pressure on the value of the domestic currency, and also the possibility of a mass withdrawal of the investments among others are threats for economic stability.

Having an adequate and sufficient reserve buffer can be an effective solution to this looming problem, but the reserves currently held cannot be regarded as sufficient. In an interview with the Deputy-Governor of the Bank of Ghana, he admitted the overstretching of the central bank's forex exchange reserve. Also, he mentions that the nation's imports supersede its export potential (Citi 97.3 FM, 2014). This compounds the pressure on the central bank's foreign exchange reserves.

According to an IMF Article IV Consultation Report (2017), both FDI and external debts have contributed to a substantial increase in external liabilities from 23% of GDP IN 2006 to 70% of GDP in 2015. Ghana has also accepted reforms to liberalize its capital account by gaining access to the International capital market in 2007. These make Ghana more open to the external world, and therefore distortions to the domestic economy should be expected.

### 2.4 The central bank of Ghana's foreign exchange reserve strategies

#### 2.4.1 The objective of the bank

The central bank of Ghana is an inflation targeting bank, though it has persistently failed to meet the inflation target bandwidth of 8 ± 2 (Article iv consultative Article IV Consultation Report, 2017, Bawumia et al., 2008; Quartey and Afful-Mensah, 2014). According to the IMF consultation Article IV Consultation Report, 2017, Ghana's inflation targeting framework has had a mixed performance. This was partly due to fiscal dominance and foreign exchange intervention constantly undermining the transmission of monetary policy (Article iv consultative Article IV Consultation Report, 2017). Assessing how the central bank, therefore, can achieve fiscal and monetary independence in the wake of high macroeconomic instability could be the way forward for the central bank.

#### 2.4.2 Central banks independence and the significance of holding reserves

Discussing the ways through which a central bank can lose its independence, massive capital flights into a country and the integration of an economy into the global financial system can rob a central bank of its command on monetary and fiscal policies (Frenkel, 1976).

One monetary policy tool that could avert the harmful effects of these factors is the holding of adequate reserves (Obstfeld et al., 2005, Popper et al., 2011 and Aizenman et al., 2010, 2011).

Steiner (2013) observed that it is impossible to achieve monetary independence in an open economy with a floating exchange regime either de facto or de jure. Having adequate reserves gives leeway for an independent monetary and financial policy despite the theory of classic trilemma (Steiner, 2013, Aizenman et al., 2010, 2011). Also, accumulating adequate reserves could be a substitute for capital control mechanisms, and can be used to absorb the net effect of capital flight, and also preserve the monetary and fiscal independence of the central bank from vulnerabilities associated to shocks in external factors. Steiner (2013) summarizes the purpose of keeping reserve as reducing the interdependence of an open economy from developments in the rest of the world.

#### 2.4.3 Comparison of Ghana's reserves to empirical standards, benchmarks or metrics

**Figure 5:**
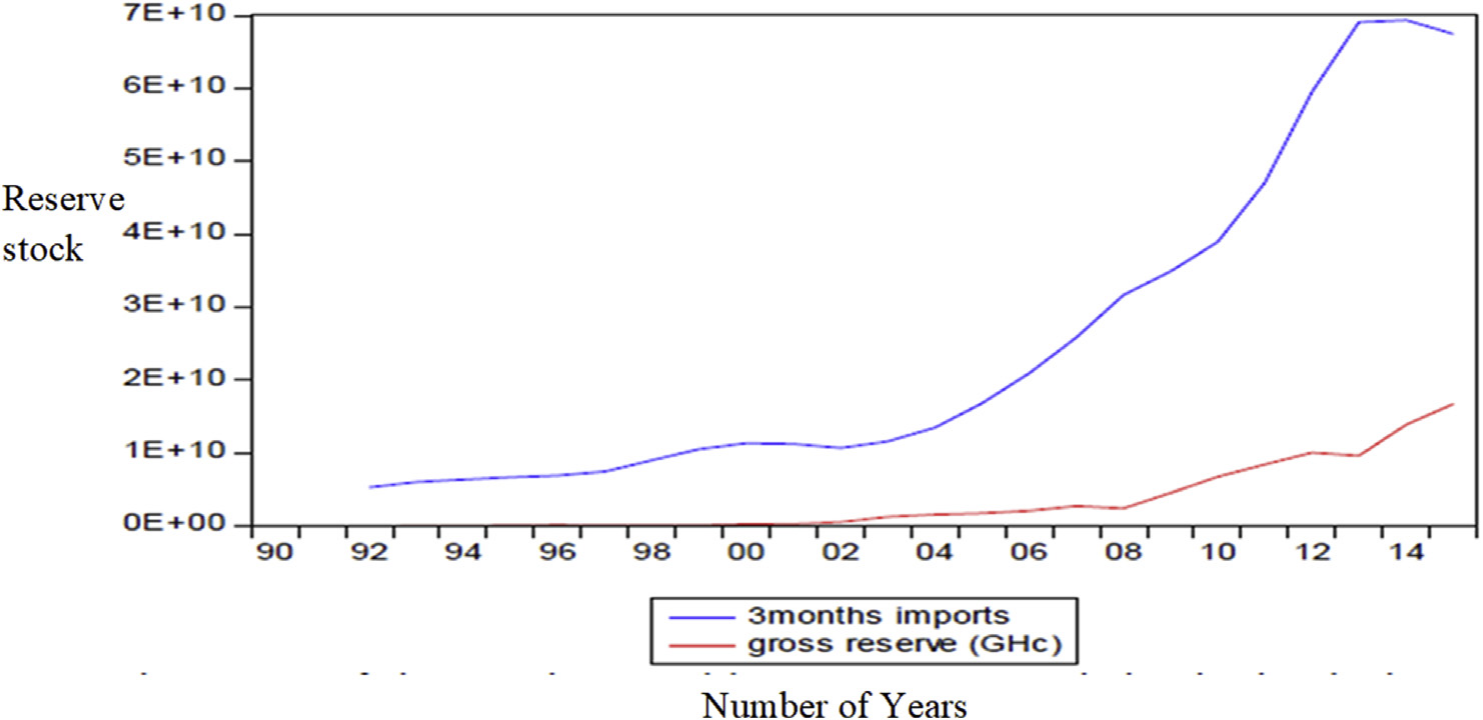
Ghana's reserves lower than the Imports benchmarks. Source: Yao and Abdul Rashid (2018), Permission for reuse of Figure Granted.

**Figure 6:**
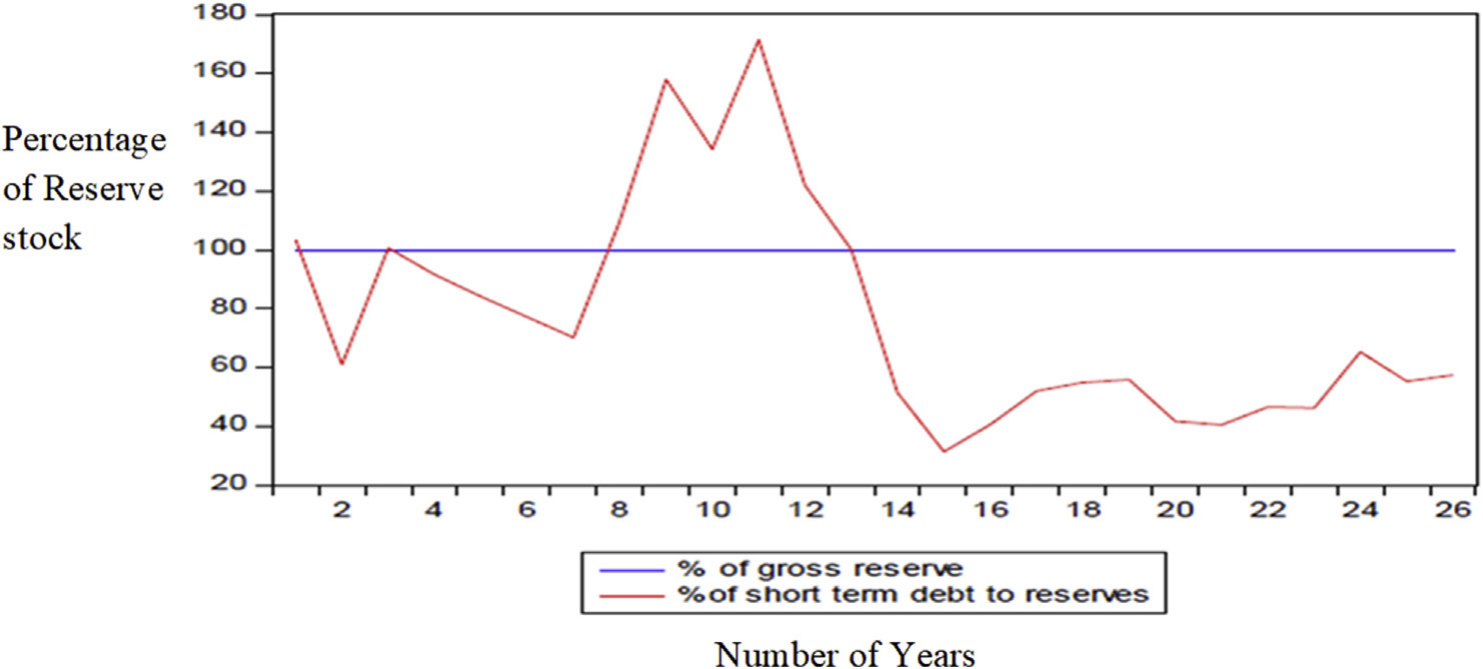
Ghana's reserves well above the Greenspan rule after 2006. Source: Yao and Abdul Rashid (2018). Permission for reuse of Figure Granted.

A casual analysis of the data using three empirical benchmarks in literature seems to point at Ghana's preference of targeting domestic shocks rather than external shocks. Ghana's reserve levels for the past two decades are well above the Greenspan rule and the twenty percent M2 benchmarks, which mostly reflects resistance to shocks in the domestic financial system. The plots show that the Central Bank of Ghana wishes to protect against the possibility of not meeting short-term debts as well as brace itself for possible capital flight. This is a combination metric where the central bank combines two general metric (i.e., the Greenspan-Guidotti rule, and the ratio of reserves to M2 rule) to stabilize the economy. This was the approach of Wijnholds and Kapteyn (2001). Figure 5 and Figure 6 show the plots of Ghana's reserves level to different empirical benchmarks, that is, reserves to import growth metrics and the reserves to short-term debts metric respectively.

Ghana since gaining access to the international capital market in 2007, and the discovery of oil in commercial quantities in 2009, has attracted massive capital inflows into the country (Article iv, consultation Article IV Consultation Report, 2017). Foreign direct investments (FDI) have mainly increased as well, and this has been a growing trend in developing countries compared to developed economies (Antwi et al., 2013). FDIs and external debts have contributed to a substantial increase in external liabilities from 26% of GDP in 2006 to 70% of GDP in 2015 (Article IV Consultation Report, 2017). Ghana's vulnerability and dependence on external economies are getting worse day by day. These threaten the monetary and fiscal independence of the Central bank. Therefore the need to brace the economy for capital flight has become increasing eminent.

The literature has grouped foreign exchange reserves and the purpose of holding them into two orderly classes. The traditional literature on foreign exchange reserves argues that reserves are to correct imbalances in the balance of payment account of economies with fixed exchange rates (Krugman, 1979; Flood and Garber, 1984; Broner, 2008). In these Papers, the level of reserves determines the duration of an unsustainable exchange rate peg (Pina, 2015).

**Figure 7:**
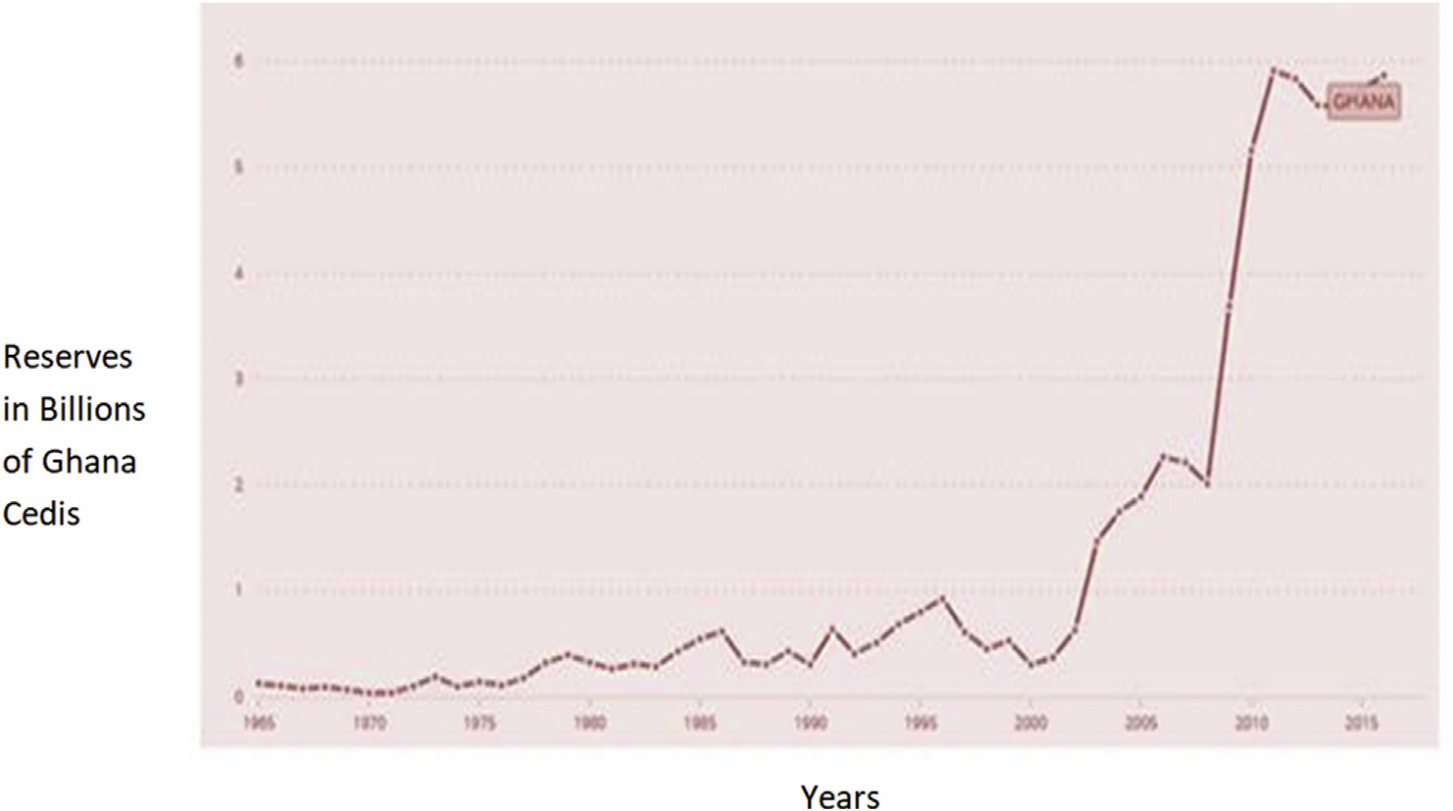
TREND in Ghana's Total Reserves (including Gold) consistent with reserve accumulation in most developing countries. Source: IMF, IFS data. Permission for reuse of Figure granted.

Recent literature views the holding of reserves as insurance or a precautionary saving against financial crisis (Alfaro and Kanczuk, 2009; Durdu et al., 2009; Jeanne and Ranciere, 2011; Bianchi et al., 2014). This group of researchers sees the stock of reserves as a buffer to be used to smooth aggregate consumption and avoid volatility in the economy. Greenspan (1999) argue that the level of reserves should be precisely equal to the level of short term debts, and therefore the ratio of reserves to short term debts should be equal to one(1). This is latterly known as the Greenspan rule (see figure 6). Figure 7 shows the trend in Ghana's reserve growth similar to other developing nations (Pina, 2015). This trend is now the order in the economies of most developing and emerging nations which is spearheaded by China's unflinching tendency to accumulate more reserves relative to the developed worlds. This astronomical growth in the reserves of the central bank of Ghana begs the question; ‘for what motives are the reserves kept and whether there exists a perpetual and acceptable pattern in the accumulation process in literature?’

## 3 Set-up and estimation of the empirical model

### 3.1 Set-up of the model

Consider a Vector Error Correction (VEC) model with seven variables below:

(1) $$\Delta \text{Xt }=\;\alpha \beta_{1}{\text{X}}_{\text{t}-1}\;+\overset{p-1}{\underset{i=1}{\sum }}{\text{ Г}}_{\text{i}}\Delta {\text{X}}_{\text{t}-\text{i}}+{\text{ U}}_{\text{t}}$$

Where.

${\text{X}}_{\text{t}}$ is a vector of endogenous variables.
${\text{β}}_{1}$Is a vector of parameters for the error correction term.
α is a vector of long-run adjustments.
${\text{Г}}_{\text{i}}$ the number of cointegrating equations
${\text{U}}_{\text{t}}$ vector of error terms

${\text{X}}_{\text{t}}$ consists of Broad Money (M2), Gross International Reserves, Short-term Debts, Exchange rates (GHc/US$), Foreign Direct Investments, Petro prices, and Cocoa prices.

We used Broad Money, and Short-term Debts in the research as measures of policy, whiles Reserves, Exchange rates, Foreign Direct Investments (FDI), Petroleum prices and Cocoa prices are measures for economic conditions.

The variables; Foreign Direct Investments (FDI), Petroleum prices and Cocoa prices, which are some of the variables used as proxies for the economic condition of the country, are either more aligned to trade or to investments in the country. These two factors are found to exert significant influence on the country's reserves stock (Government of Ghana, 2014).

The hypotheses below were therefore formulated to test whether Ghana's reserves stock are in line with any empirical benchmarks, and also to examine the strategy of reserves accumulation by the central bank of Ghana.

H1 Short-run changes in foreign exchange reserves of the central bank is equal to 20% of the money supply (M2)

$$b_{ij}=0.2$$

Where.

‘b’ is the short-run beta coefficients of money supply in the reserve model and ij give the position with respect to elements i and j, respectively.

H2 Short-run changes in foreign exchange reserves of the central bank is equal to the changes in short-term debts

$$b_{ij}=1$$

Where.

‘b’ is the short-run beta coefficients of short-term debts in the reserve model and ij give the position with respect to elements i and j, respectively.

H3 The impulse responses of the central bank of Ghana's reserves adjust properly in line with the predictions of empirical findings in research.

H4 Exchange Rate depreciation contributes to increase Reserves build-up of the Central Bank of Ghana.

The use of the vector regression models in this sort of analyses will help to discover the dynamics and impulse responses of the reserves stocks to shocks in the metric variables. This will help in explaining whether the reserve level is as a result of a policy change or it is just an endogenous response to changes in the economy.

### 3.2 The data

The data for the research is a monthly time series data taken from the Central Bank of Ghana database and from the World Bank database from 1990 to 2017. We collected data on gross reserves, foreign direct investments, petroleum prices, cocoa prices, exchange rates, broad money (M2), and short-term debts.

The Petroleum prices are the world Brent crude oil spot prices in U.S dollars per barrel, and the Cocoa prices are the indicative cocoa prices in U.S dollars per barrel. Note that the commodity prices are in nominal values, and also the exchange rates are the Inter-Bank End Month exchange rates in U.S dollars.

Additionally, we defined M2 as the sum of M1 plus time and savings deposits as well as foreign currency deposits. Note that M1 is the currency held by the public and also the demand deposits with banks. Reserves, on the other hand, are the external assets that are readily available to and controlled by monetary authorities for meeting the balance of payments financing needs. This includes gold, special drawing rights (SDRs), reserve position in the International Monetary Fund (IMF), and other reserve assets.

The Short term debts are the debts with an original maturity of one year or less, whiles Foreign Direct Investments (FDIs) is the net investment inflows which give investors a lasting management interest in an enterprise operating in an economy other than that of the investor.

The choice of these research variables hypothesized to affect the reserves stock of the central bank of Ghana is deeply grounded in theory. Most of the empirical studies, as we have already referenced in the literature, have found significant relationships between the research variables. Lane and Burke (2001) and Bussière et al. (2015) have found money supply and short-term debts as essential determinants of the level of reserves held by central banks. Also, foreign capital inflows and the eventual risk of sudden withdrawal of these capitals is another essential factor in determining the amount of international reserves that a central bank should keep (Steiner, 2013). This particular scenario is especially real in an open economy such as Ghana; where the use of capital control measures to limit external exposures is restricted.

Furthermore, many empirical studies have found growing reserve accumulation to be predominant in economies that are rich in natural resources and are mostly producers of primary raw materials (Pina, 2015; ARA, 2013). According to IMF Article IV Consultation Report (2017), most of the inflows from the proceeds of primary export commodities such as cocoa, and now petroleum are among the factors that influence the international reserves of Ghana. Therefore, the decision to include these variables in the research is purely theoretical and backed by empirical underpinnings.

### 3.3 Unit root test

Before testing the empirical model, a test of unit root stationarity on the variables is important in other to determine how the variables enter into the general model.

The general regression form below is used for the standard Unit root test:

(2) $$\Delta \text{Xt }=\alpha +(\beta -1){\text{X}}_{\text{t}-1}\;+\overset{P-1}{\underset{i=1}{\sum }}\Gamma \text{i}\Delta \text{Xt}-\text{i }+\text{ Ut }$$

#### 3.3.1 Decision rule

H_0_: β = 1; There is a Unit Root.

H_A_: β < 1; There is no Unit Root.

At the critical value of 10%, the ADF- test has found all the series to be nonstationary I(1) except for Petroleum prices (See Table 1 and Table 2). Based on this conclusion, the variables entered in the empirical model in first difference.

**Table 1:** Augmented Dickey-Fuller test of Unit.

Series name	REGRESSION ESTIMATES						ADF Test		
	Constant		Lag Regressors		Trend				
	t- stat	Prob.	t- stat	Prob.	t-stat	Prob	t-stat	10% C. Value	Prob.
Gross reserves	-0.396	0.6924	-2.096	0.0369	1.8793	0.0611	-2.096	-3.1347	0.5457
M2	-0.1808	0.8566	4.4395	0.0008	0.3181	0.7506	4.4395	-3.1347	1
Short-term debts	-1.0561	0.2917	-0.089	0.9293	1.8476	0.0656	-0.354	-3.1347	0.9949
FDI	-1.1436	0.2536	-0.354	0.7236	1.9524	0.0517	-0.354	-3.1347	0.9888
ExRate	-0.6976	0.4859	0.15	0.8808	1.3102	0.191	0.15	-3.1347	0.9975
PetroP	0.6398	0.5227	-4.003	0.0001	3.5092	0.0005	-4.003	-3.1347	0.0095
CocoaP	2.3588	0.0189	-2.781	0.0057	2.2763	0.0235	-2.781	-3.1346	0.2052

Decision rule: Reject the null hypothesis when p-value is less than or equal to 0.10 (10%) significance level.

**Table 2:** Summary of the ADF test statistics.

Series name	ADF Test Outcome	
	Series in Level form	Series in First Difference form
Gross reserves	Has unit root	Has no unit root
M2	Has unit root	Has no unit root
Short-term debts	Has unit root	Has no unit root
FDI	Has unit root	Has no unit root
ExRate	Has unit root	Has no unit root
PetroP	Has no unit root	N/A
CocoaP	Has unit root	Has no unit root

N/A: Series already stationary.

Also, the variable CocoaP has a significant constant value, and all the variables have a significant trend except for M2 and ExRate. We, therefore, impute a constant and a trend in the long-run model.

### 3.4 Johansen cointegration test

We run a cointegration test with the six variables that are I(1). The general regression equation to test cointegration for VAR models is:

(3) $$\Delta Xt=\overset{p}{\underset{i=1}{\sum }}\pi_{i}X_{t-1}+\;U_{t}\;\text{, where}{\text{ u}}_{\text{t}}∼\text{IN[0,}\Omega \text{].}$$

Where.

X_t_ is a (6 × 1) vector of I(0) variables.

When the series of the regression X_t_ is I(1), the model is reformulated to include an error correction term (Engle and Granger, 1987; Johansen, 1988).

(4) $$\Delta \text{Xt }=\alpha \beta {\text{X}}_{\text{t}-1}+\overset{p-1}{\underset{i=1}{\sum }}\Gamma_{\text{i}}\Delta {\text{X}}_{\text{t}-\text{i}}+U_{t}$$

As we have already mentioned, for the system in Eq. (2) to be correctly specified, the residuals from the linear combination of the I(1) variables (αβX_t-1_) must be I(0).

We have run the cointegration test in this paper using both Trace test and the Maximum Eigenvalue test from the Johansen test methodology in Table 3. These tests showed different test results; that is, the Trace test showed two cointegration equations whiles the Maximum Eigenvalue test showed only one cointegration equation.

**Table 3:** Results from cointegration test.

Hypothesis	Trace Test	Maximum Eigenvalue Test
r = 0	178.7676 (0.0000)	83.4430 (0.0000)
r = 1	95.3245 (0.0156)	33.8057 (0.1512)
r = 2	61.5188 (0.0771)	-

Note: p-value in parentheses (). The asymptotic p-values are estimated with no constant and but a trend in the cointegrating regression.

The Johansen test rejected the Maximum Eigenvalue test for (r=1) in Table 3. This is why the second rank (r=2) of the cointegration test for the Maximum Eigenvalue test in Table 3 is not shown. We will be continuing this paper with the results of the Trace test statistics.

Remember that none of the nonstationary series I(1) has a significant constant in the unit root table except Cocoa prices. We, therefore, compute the Johansen cointegration test without a constant in the cointegration equation.

(5) $$\Delta \text{Xt }=\;\alpha \beta_{1}{\text{X}}_{\text{t}-1}\;+\overset{p-1}{\underset{i=1}{\sum }}\;\Gamma_{\text{i}}\Delta {\text{X}}_{\text{t}-\text{i}}+U_{t},$$

The normalized cointegration equations from the unrestricted long-run relationship of the I(1) variables are:

(6) CE (1)_t_ = Gross_reserves -9885.471*ExRate +6.3269*M2 + 2.2323*CocoaP – 1.42E-05*FDI +7.73E-06*Stdebts – 26.1458*@Trend

(7) CE (2)_t_ = exrate –0.0003*M2 – 0.0004*CocoaP +1.09E-09*FDI -1.05E-09*Stdebts +0.0005*@Trend

Table 4a, b shows the restricted cointegration relationship and the significance value of the long-run restrictions.

**Table 4a:** Test of long-run relationship.

Cointegration Restrictions:										
B(1,1) = 1, B(1,3) = 0, B(1,4) = -1, B(2,2) = 1, B(2,1) = 0, B(1,5) = 1, B(1,6) = 0, B(1,7) = 1, B(1,2) = 1, A(2,1) = 0, A(2,2) = 0, A(7,1) = 0, A(7,2) = 0										
Maximum iterations (500) reached.										
Restrictions identify all cointegrating vectors										
LR test for binding restrictions (rank = 2):										
Chi-square(9)					13.82355					
Probability					0.128742					
	Gross_R	PetroP	Stdebts	ExRates		M2	FDI	CocoaP	@Trend	Constant
	1.000	1.000	0.0000	-1.000		1.000	0.000	1.000	-10.27646	-9434.57
Std error									(8.7397)	
t-value									[-1.1758]

**Table 4b:** Short-Run Hypotheses testing.

Statement of Hypothesis	WALD TEST	
	Chi-Square	p-value
$H_{1}$: Short-run changes in foreign exchange reserves of the central bank is equal to 20% of money supply (M2) $b_{t-i}=0.2$	71.1336	0.0000
$H_{2}$: Short-run changes in foreign exchange reserves of the central bank is equal to the changes in short-term debts $b_{t-i}=1$	16.9363	0.0308
$H_{4}$: Exchange Rate depreciation contributes to Reserves build-up of the Central Bank of Ghana. $b_{t-i}=-1$	74.0934	0.0000

### 3.5 Model estimation

We have conducted a unit root test and also a cointegration test on the series identified to be I(1). We, therefore, set-up a VEC model in Eviews 9.0 using seven variables, an intercept and a trend in the cointegration equations (CE), and no trend in the VAR model. All the models in the VEC have a lag length of eight months. This was done through the estimation of different VAR lags, and selecting the VAR lag that make the residuals free of autocorrelation (Hendry and Juselius, 2001). No lag length information criterion was specifically used. The output of the VEC is shown in Appendix A.

We also imposed several restrictions on the beta and alpha coefficients of the long-run equations during the estimation period. Note that the long-run equations were normalized to reserves and petroleum prices respectively, whiles we further assumed that exchange rate depreciation leads to positive reserves build-up by the central bank of Ghana (Pina, 2015; Aizenman and Sun, 2012). Additionally, we also assumed that except for the level of short-term debts and foreign direct investment, all other variables contribute to the long-run response of the normalized reserve equation. What this assumption means, however, is that we can take away short-term debts and foreign direct investments from the long-run relationships of the normalized reserve equation. This long-run restriction is as an alternative test for the Greenspan-Guidotti rule.

Furthermore, we used commodity prices (i.e., petroleum prices and cocoa prices) in the model as weakly exogenous variables. This was tantamount to assuming that changes in commodity prices were not affected by the adjustments in the long-run model and that all the adjustments are made by the other variables in the model (see Table 4a for the test of these long-run assumptions).

Lastly, some hypotheses testing and restrictions on the short-run coefficients of some selected variables were estimated and displayed in Table 4b.

**Figure 8:**
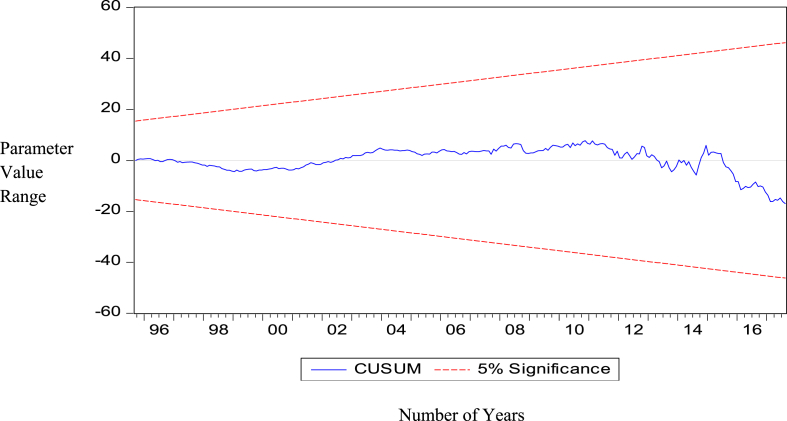
Parameters Stability test.

The Appendix A shows the output of the vector error correction model (VECM), and Table 5 shows the model's residual diagnosis test. Table 6 also shows the results of the Wald-test joint test of significance of the short-run coefficients together with the confidence intervals of the point estimates of the reserve model. Figure 8 also shows the parameter stability test of the reserve model using CUSUM test Recursive Estimates. This shows the consistency of the results and estimates of the reserve model.

**Table 5:** Residual diagnostic test.

Residual Diagnosis test	Measurement Statistic	Prob. (p)	DECISION RULE:	RESULTS
Test of Serial correlation	6.8806	0.5496	$H_{0}$: IF p≤0.05, reject the $H_{0}$ of no serial correlation, otherwise accept the $H_{0}$	No serial correlation
Test of Heteroscedasticity (Chi-square)	13.0290	0.1109	$H_{0}$: IF p≤0.05, reject the $H_{0}$ of no heteroscedasticity, otherwise accept the $H_{0}$	No heteroscedasticity
Test of Normality (Jarque Bera Test)	842.9854	0.0000	$H_{0}$: IF p≤0.05, reject the $H_{0}$ of normality, otherwise accept the $H_{0}$	Not normally distributed

**Table 6:** Short-run Joint Test of significance of Variables in the Reserve model.

Test of Independent Variables in the RESERVE EQUATION	SHORT-RUN COEFFICIENTS DIAGNOSIS/WALD-TEST COEFFICIENT RESTRICTIONS		95% CONFIDENCE INTERVAL (CI)		POINT ESTIMATES OF VARIABLES
	CHI-SQUARE VALUE	PROBABILITY	LOW	HIGH	
Grosss Reserves	63.3543	0.0000	-0.337084	-0.076685	-0.206885
			-0.171434	0.110050	-0.030692
			-0.382690	-0.086179	-0.234435
			-0.142856	0.144410	0.000777
			-0.170428	0.126427	-0.022000
			-0.107154	0.190384	0.041615
			-0.443431	-0.143628	-0.293530
			-0.202689	0.087399	-0.057645
Petroleum	11.3609	0.1821	-4.517044	6.913549	1.198253
			-4.100624	7.344608	1.621992
			-1.810361	9.799999	3.994819
			-2.286843	9.225678	3.469418
			-13.50262	-1.846159	-7.674390
			-2.851886	8.949451	3.048782
			-4.427105	7.303919	1.438407
			-5.732783	5.860316	0.063767
Short-term debts	16.9363	0.0308	-4.14E-07	-5.24E-08	-2.33E-07
			-4.34E-07	-4.84E-08	-2.41E-07
			-1.59E-07	2.43E-07	4.18E-08
			-2.55E-07	1.47E-07	-5.40E-08
			-1.72E-07	2.35E-07	3.13E-08
			-3.08E-07	1.07E-07	-1.00E-07
			-2.58E-07	1.02E-07	-7.81E-08
			-3.22E-07	7.70E-09	-1.57E-07
Exchange rates	74.0684	0.0000	-368.3304	362.7084	-2.811007
			-314.2141	500.6504	93.21819
			-1637.181	-779.1298	-1208.155
			-1266.163	-296.4698	-781.3164
			-58.23702	898.4444	420.1037
			-80.99924	867.5370	393.2689
			127.1367	1073.572	600.3543
			124.8323	1052.522	588.6773
M2	20.4968	0.0086	0.015311	0.100988	0.058149
			0.023179	0.114511	0.068845
			0.001148	0.111119	0.056133
			0.000887	0.127563	0.064225
			0.017402	0.162967	0.090185
			0.007428	0.219470	0.113449
			-0.079698	0.119370	0.019836
			-0.114229	0.064706	-0.024762
FDI	30.7151	0.0002	-1.84E-07	5.87E-08	-6.27E-08
			-2.08E-07	3.95E-08	-8.44E-08
			-4.17E-07	-1.69E-07	-2.93E-07
			-3.88E-07	-1.25E-07	-2.56E-07
			-3.17E-07	-4.69E-08	-1.82E-07
			-2.24E-07	5.40E-08	-8.49E-08
			-2.10E-07	4.58E-08	-8.23E-08
			-8.99E-08	1.49E-07	2.95E-08
Cocoa Prices	6.5901	0.5814	-0.211631	0.153411	-0.029110
			-0.150542	0.208412	0.028935
			-0.225793	0.136325	-0.044734
			-0.070824	0.280373	0.104775
			-0.286983	0.060466	-0.113259
			-0.288879	0.060574	-0.114153
			-0.128128	0.220345	0.046109
			-0.265200	0.080723	-0.092239

NOTE: The point-by-point estimation of the variables are all within the confidence bands which is an indication of the model's stability.

## 4 Impulse response

**Figure 9:**
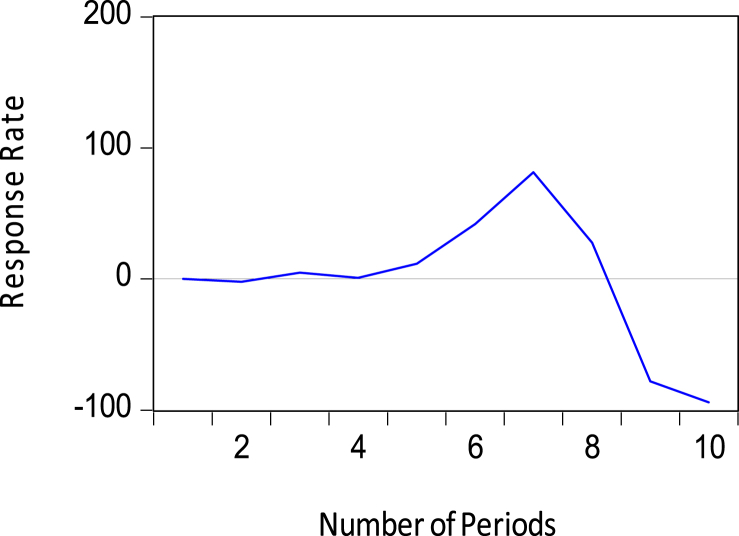
Response of foreign exchange reserves to M2.

**Figure 10:**
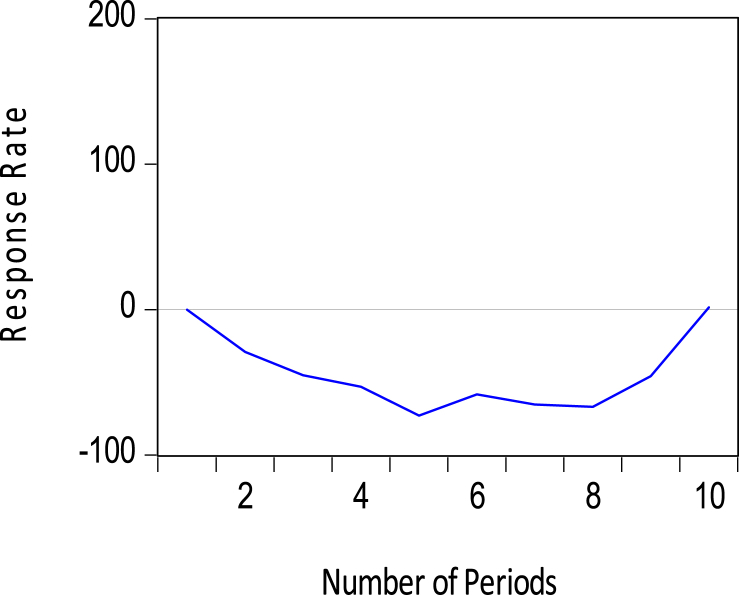
Response of foreign exchange reserves to Short-term debts.

Short-term movements in the policy variables (that is, M2 and Short-term Debt) jointly impact the amount of reserves, however in different ways. Whereas a positive shock to the M2 policy variable leads to an upward adjustment in reserves in Figure 9, a positive shock to Short-term debt causes a downward adjustment to the stock of reserves as seen in Figure 10. Note that Whiles the former is expected, the latter is inconsistent with the literature on Foreign Exchange reserve accumulation. These short-term impacts of the money supply and short-term debts on the central bank's reserves growth were found to be statistically significant and within the confidence bands in Table 6.

**Figure 11:**
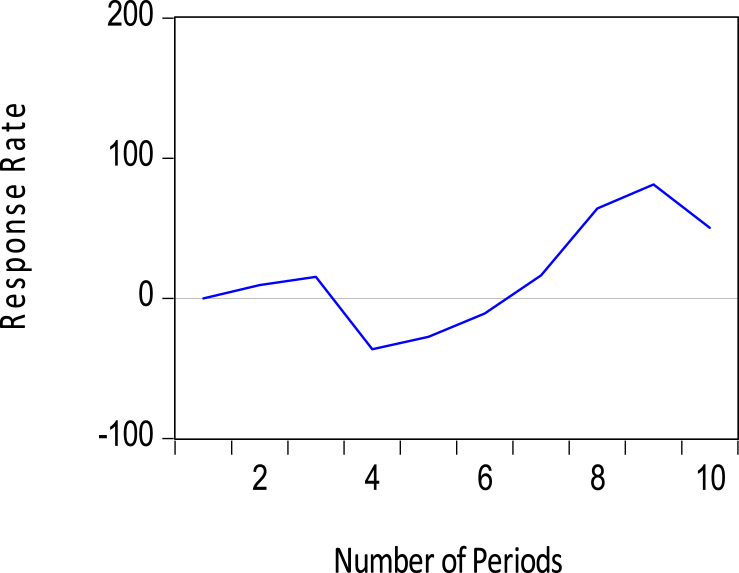
Response of foreign exchange reserves to exchange rates.

Also, Short-run appreciations in exchange rates (positive shock to the value of the cedi) impacts positively on reserve accumulation, and the effect begins to deter off after three months. Figure 11 shows the impulse response of the reserves to positive shocks in the exchange rates. Again, this response was statistically significant in the output of the Wald test in Table 6.

**Figure 12:**
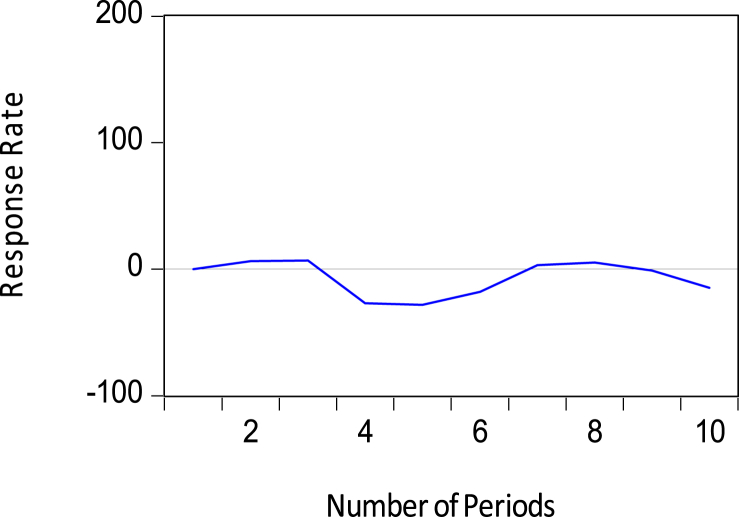
Response of foreign exchange reserves to FDI.

Additionally, short-run shocks to FDIs lead to a positive impact on reserves for the first three months in Figure 12. This result is consistent with the use of reserves as a buffer against external shocks and capital flight.

**Figure 13:**
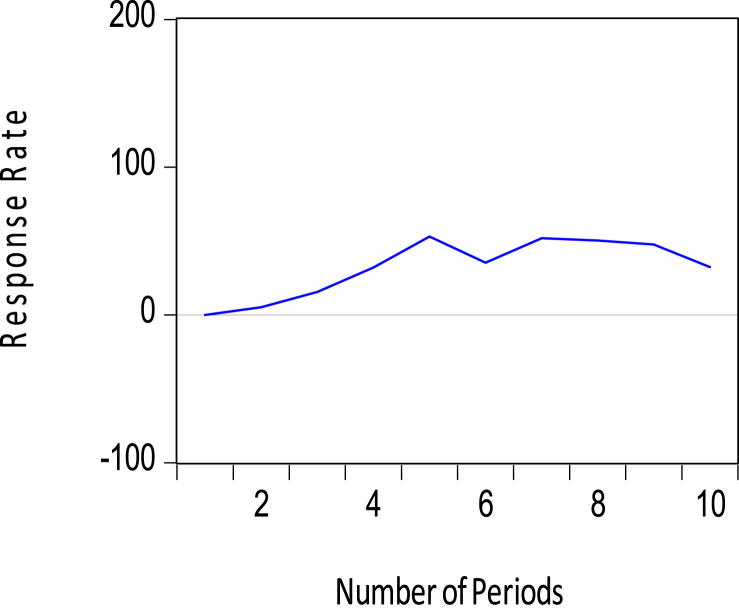
Response of foreign exchange reserves to petroleum prices.

**Figure 14:**
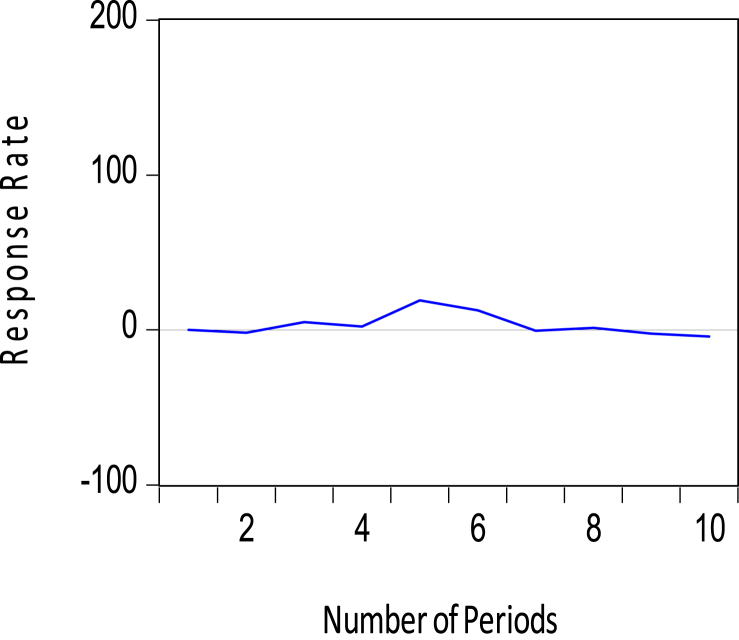
Response of foreign exchange reserves to cocoa prices.

Furthermore, the positive response of reserves to shocks in trade variables such as Petroleum prices and Cocoa prices in Figure 13 and Figure 14 were found to be statistically insignificant on the short-run movements of the central bank's foreign exchange reserves. There is however the possibility that these results are because of the use of unit prices instead of total revenue from the production of these two commodities.

## 5 Variance decomposition

Besides own shocks, government short-term debts and exchange rates respectively lead in explaining the variations in government reserves within the first three months of positive shocks to economic variables. Given the fact that Ghana's short-term debts are mostly from external borrowings and also that the exchange rate is more influenced by external pressures, we; therefore, conclude that the variations in the reserve stock of Ghana's central bank are explained mostly by external forces and pressures in the short-run.

However, in the long-run (i.e., periods from six months onwards) the impact of short-term debts, money supply, exchange rates and petroleum prices become the essential factors in explaining the variations in the reserves, though the significance of money supply and exchange rates respectively and to be precise become the more dominant by the passage of time. This evidence shows that the long-run variations in the reserves of the central bank of Ghana are due mostly to developments in the financial sector and partly due to external pressures or forces. Also, the growing influence of money supply and the exchange rates in explaining the changes in reserves point further to the central bank's preference to adapting the reserves stock to the growths in the financial sector and the perceived directional movements of the exchange rates.

Using the central bank's reserves to support and stabilize the financial sector is the duty of most central banks. In recent times, the central bank of Ghana has roll-out the Emergency Liquidity Assistance program (ELA). The objective of this program was to inject liquidity into the banking sector and position the banks to be able to fulfill their core mandate of extending private sector credit to strategic sectors of the economy. However, the failure of the ELA program has led to excess liquidity in the financial sector and has created risk in the balance sheet of the central bank (Article iv Article IV Consultation Report, 2017). Also, in the recent Bank sector Article IV Consultation Report (2017), stabilizing the financial sector has become a significant policy issue and similar calls from the IMF in the article iv Article IV Consultation Report (2017) is emphasized. This according to the IMF is needed for the effective transmission of Ghana's policy variables through the banking system (Article iv Article IV Consultation Report, 2017).

Table 7 shows the explanatory power of economic and policy variables in the variations of the Central Bank's reserves over specify time ranges. It also gives recommendations for the specification of a forecasting model. The analysis in the table suggests that a univariate ARIMA model should be used to forecast foreign exchange reserves for periods within three months, and a reduced-form VAR model containing money supply, exchange rates, short-term terms and petroleum prices for periods beyond three months.

**Table 7:** Variance decomposition and choice of forecasting Model for Foreign Exchange Reserves in Ghana.

TIME RANGE	HIGHEST EXPLANATORY REGRESSOR		INTERPRETATION OF EXPLAINED VARIANCE	RECOMMEDED MODEL SPECIFICATION FOR FORECASTING RESERVES
	Variable Name	Degree of variance Explained		
Time within three months	FX Reserves	94.9526	Own shocks more Influential than any Variable in the model	Use Univariate ARIMA models to Forecast reserves within this time range after shocks to policy and economic variables.
Time within one year	FX Reserves	53.9195	Own shocks more Influential than any Variable in the model	Univariate ARIMA models not sufficient (Use Reduce-Form VAR, Including FX Reserves and M2, short-term debts, exchange rates and petro prices in regressors)
Time from One to three years	Broad Money (M2)	50.2460	Other variables more Influential than own Shock	Use Reduce-Form VAR (including M2 and foreign exchange reserves, short-term debts, exchange rates and petro prices in regressors)

## 6 Conclusion

The research rejected the short-run matching of the reserves stock of the central bank of Ghana to the theoretical standard metrics of 20% of broad money and 100% of short-term debts.

Also, the impulse response functions showed mixed results of the adjustments in reserves of the central bank of Ghana to the predictions of empirical theories and findings. For example, foreign exchange reserves respond negatively to positive shocks in short-term government debts. This behavior is inconsistent with conventional theories on the relationship between reserves and short-term debts as highlighted in the works of Ben-Bassat et al. (1992), Prabheesh (2013) and Guidotti and Greenspan (1999). Ghana being a net borrower and potentially vulnerable to sudden debt retirement by lenders should match the growths in government debts to the reserves build-up. However, the results of the impulse response show the reserves growth is not a planned intervention of the central bank to provide a perpetual cover for short-term debts. According to an IMF report, “whiles reserves build-up in Ghana rests on occasionally large capital inflows, a more transparent and articulately planned intervention strategy is needed to build up reserves in a sustained way” (IMF Article IV Consultation Report, 2017). This situation is likely to increase sovereign risk and make future borrowing much more difficult or expensive. Also, Alfaro and Kanczuk (2009), Durdu et al. (2009) and Bianchi et al. (2014) argued for the use of reserves as a buffer to protect and smooth domestic trade and prices. Our long-run hypotheses test has confirmed a long-run co-movement of the primary export commodity prices and reserves growth by the central bank. This result is however not supported in the short-run. This short-run outcome, however, could be as a result of the use of unit prices in the model instead of total revenue from these commodities. We, therefore, recommend further research on trade analysis using total revenues from the commodities.

There are however some impulse responses that we found to be consistent with some empirical findings in research. For instance, the stock of reserves responds positively to foreign direct investments in the first three months of a shock. This impulse response is desired especially given the growing increase of foreign investments in Ghana primarily due to the expansive operations of the Ghana Investment Promotion Centre and the discovery of oil in commercial quantities. The need for this trend to continue perpetually is significant as a buffer for sudden withdrawals of this foreign capital. Steiner (2013) asserts that reserves accumulation by some nations could be an alternative for capital control measures, and the positive response of reserves plus the significant impact it has on foreign capital could suggest an attempt at a similar strategy by the central bank of Ghana. Another promising trend in the reserve build-up is the efforts by the central bank to match the growths in reserves to the growths in the money supply. Though the test of the short-run changes in reserves to the standard metric of 20% of the money supply has failed, the test of money supply contributing to the long-run adjustments in reserves was successful. Additionally, the reserves were also found to respond significantly and in the right direction to the short-run changes in the money supply. This fully-fledged matching of the reserves, defined as having both long-run and short-run relationship with the money supply function, is an all-out strategy by the central bank to match the reserves build-up with the growths in the financial sector.

Lastly, the test of exchange rate depreciation on positive reserves build-up of the central bank was successful by the long-run test of restrictions. This conclusion is therefore in agreement with the assertion in Pina (2015) and Aizenman and Sun (2012) that some central banks may rely on the depreciation of their currencies for reserves build-up. However, the Wald-test rejected the short-run relationship of the exchange rates on reserves stock. In the model of Pina (2015), the central bank accumulates international reserves resorting to inflation. This then leads to the value of the domestic currency decreasing relative to the value of foreign currencies. What the central bank does is to use this accumulated reserves when a crisis hit the economy to finance the financial sector. This is a well-known strategy for maintaining the value of the domestic currency and preventing the rates from collapsing (Dominguez et al., 2012).

In explaining the contribution of each type of shock to the forecast error variance of the central bank's reserves, this research concludes that the short-run variations in reserves are explained mostly by external forces, whereas the long-run variations in reserves are explained mostly by developments in the financial sector and partly by external forces. Therefore, an econometric model to explain the variations in reserves for a period beyond three months must specify in the model both the financial sector and external sector variables.

## Declarations

### Author contribution statement

A. R. A. Rahaman: Performed the experiments; Analyzed and interpreted the data; Contributed reagents, materials, analysis tools or data; Wrote the paper.

Y. Hongxing: Conceived and designed the experiments.

### Funding statement

This work was supported by This work was supported by the National Natural Science Foundation of China no (71701082 and 71271103).

### Competing interest statement

The authors declare no conflict of interest.

### Additional information

No additional information is available for this paper.

## Figures and Tables

**Table:** 

Vector Error Correction Estimates							
Date: 04/21/19 Time: 23:01							
Sample (adjusted): 1990M10 2017M08							
Included observations: 323 after adjustments							
Standard errors in ( ) & t-statistics in [ ]							
Cointegration Restrictions:							
B(1,1) = 1, B(1,3) = 0, B(1,4) = -1, B(2,2) = 1, B(2,1) = 0, B(1,5) = 1, B(1,6) = 0, B(1,7) = 1, B(1,2) = 1, A(2,1) = 0, A(2,2) = 0, A(7,1) = 0, A(7,2) = 0							
Maximum iterations (500) reached.							
Restrictions identify all cointegrating vectors							
LR test for binding restrictions (rank = 2):							
Chi-square(9)	13.82355						
Probability	0.128742						
Cointegrating Eq:	CointEq1	CointEq2					
GROSS_R(-1)	1.000000	0.000000					
PETROLEUMP(-1)	1.000000	1.000000					
STDEBTS(-1)	0.000000	4.00E-08					
		(1.0E-07)					
		[ 0.38575]					
EXRATE(-1)	-1.000000	531.0640					
		(90.5296)					
		[ 5.86619]					
M2(-1)	1.000000	-0.252575					
		(0.03009)					
		[-8.39325]					
FDI(-1)	0.000000	2.48E-07					
		(4.2E-08)					
		[ 5.87626]					
COCOAP(-1)	1.000000	-0.065760					
		(0.03975)					
		[-1.65452]					
@TREND(90M01)	-10.27646	-1.364885					
	(8.73974)	(0.92859)					
	[-1.17583]	[-1.46985]					
C	-9434.578	950.8511					
Error Correction:	D(GROSS_R)	D(PETROLEUMP)	D(STDEBTS)	D(EXRATE)	D(M2)	D(FDI)	D(COCOAP)
CointEq1	0.039276	0.000000	-12118.64	-1.92E-05	0.108610	-32104.38	0.000000
	(0.00885)	(0.00000)	(8765.74)	(2.5E-06)	(0.04441)	(13293.3)	(0.00000)
	[ 4.43559]	[NA]	[-1.38250]	[-7.73942]	[ 2.44548]	[-2.41507]	[NA]
CointEq2	0.395136	0.000000	-212134.9	-0.000206	0.336905	-434009.7	0.000000
	(0.09844)	(0.00000)	(97454.2)	(2.8E-05)	(0.49376)	(147790.)	(0.00000)
	[ 4.01385]	[NA]	[-2.17677]	[-7.47277]	[ 0.68232]	[-2.93666]	[NA]
D(GROSS_R(-1))	-0.206885	0.000609	451910.4	2.03E-05	-0.236550	738724.9	0.060744
	(0.06612)	(0.00147)	(64968.5)	(1.8E-05)	(0.32837)	(98643.0)	(0.04599)
	[-3.12869]	[ 0.41403]	[ 6.95583]	[ 1.10401]	[-0.72037]	[ 7.48888]	[ 1.32075]
D(GROSS_R(-2))	-0.030692	-0.001658	374228.6	4.61E-05	0.763177	451492.7	-0.053642
	(0.07148)	(0.00159)	(70229.3)	(2.0E-05)	(0.35496)	(106630.)	(0.04972)
	[-0.42939]	[-1.04375]	[ 5.32867]	[ 2.32068]	[ 2.15003]	[ 4.23418]	[-1.07897]
D(GROSS_R(-3))	-0.234435	0.001080	37811.00	8.11E-05	0.361351	5157.322	0.004486
	(0.07530)	(0.00167)	(73978.6)	(2.1E-05)	(0.37391)	(112323.)	(0.05237)
	[-3.11353]	[ 0.64520]	[ 0.51111]	[ 3.87659]	[ 0.96641]	[ 0.04592]	[ 0.08565]
D(GROSS_R(-4))	0.000777	0.003154	150514.8	1.45E-05	-0.156998	166590.3	-0.054417
	(0.07295)	(0.00162)	(71671.9)	(2.0E-05)	(0.36225)	(108821.)	(0.05074)
	[ 0.01066]	[ 1.94481]	[ 2.10005]	[ 0.71347]	[-0.43340]	[ 1.53087]	[-1.07253]
D(GROSS_R(-5))	-0.022000	0.002614	142394.4	2.89E-05	-0.231495	231476.1	-0.137342
	(0.07538)	(0.00168)	(74064.3)	(2.1E-05)	(0.37434)	(112453.)	(0.05243)
	[-0.29185]	[ 1.56019]	[ 1.92258]	[ 1.37919]	[-0.61840]	[ 2.05842]	[-2.61949]
D(GROSS_R(-6))	0.041615	0.003913	77894.95	3.06E-05	0.205894	266237.4	0.032268
	(0.07556)	(0.00168)	(74234.6)	(2.1E-05)	(0.37520)	(112712.)	(0.05255)
	[ 0.55078]	[ 2.32950]	[ 1.04931]	[ 1.45603]	[ 0.54875]	[ 2.36211]	[ 0.61403]
D(GROSS_R(-7))	-0.293530	0.002740	-64807.23	1.35E-06	-0.060248	21360.37	0.031151
	(0.07613)	(0.00169)	(74799.7)	(2.1E-05)	(0.37806)	(113570.)	(0.05295)
	[-3.85558]	[ 1.61910]	[-0.86641]	[ 0.06379]	[-0.15936]	[ 0.18808]	[ 0.58830]
D(GROSS_R(-8))	-0.057645	0.000489	-16404.39	1.83E-06	0.073882	62150.14	-0.032008
	(0.07366)	(0.00164)	(72376.0)	(2.0E-05)	(0.36581)	(109890.)	(0.05124)
	[-0.78254]	[ 0.29841]	[-0.22666]	[ 0.08945]	[ 0.20197]	[ 0.56557]	[-0.62472]
D(PETROLEUMP(-1))	1.198253	0.146073	-1395361.	0.000117	9.927084	-5123910.	-2.822202
	(2.90265)	(0.06452)	(2851893)	(0.00081)	(14.4143)	(4330082)	(2.01887)
	[ 0.41281]	[ 2.26383]	[-0.48928]	[ 0.14558]	[ 0.68870]	[-1.18333]	[-1.39791]
D(PETROLEUMP(-2))	1.621992	0.108352	1376483.	-0.001175	2.173538	-8289911.	1.851799
	(2.90637)	(0.06461)	(2855545)	(0.00081)	(14.4328)	(4335627)	(2.02146)
	[ 0.55808]	[ 1.67708]	[ 0.48204]	[-1.45498]	[ 0.15060]	[-1.91204]	[ 0.91607]
D(PETROLEUMP(-3))	3.994819	0.055295	-1782044.	-0.001764	-0.904888	-2691997.	1.686148
	(2.94830)	(0.06554)	(2896744)	(0.00082)	(14.6410)	(4398180)	(2.05062)
	[ 1.35496]	[ 0.84369]	[-0.61519]	[-2.15403]	[-0.06180]	[-0.61207]	[ 0.82226]
D(PETROLEUMP(-4))	3.469418	-0.160548	-3385823.	-0.000352	7.120018	-6519616.	-3.287109
	(2.92346)	(0.06499)	(2872334)	(0.00081)	(14.5177)	(4361117)	(2.03334)
	[ 1.18675]	[-2.47046]	[-1.17877]	[-0.43374]	[ 0.49044]	[-1.49494]	[-1.61660]
D(PETROLEUMP(-5))	-7.674390	-0.053214	-3747830.	-0.001050	1.619594	-11800696	-1.730815
	(2.96001)	(0.06580)	(2908246)	(0.00082)	(14.6992)	(4415644)	(2.05877)
	[-2.59269]	[-0.80873]	[-1.28869]	[-1.27726]	[ 0.11018]	[-2.67247]	[-0.84070]
D(PETROLEUMP(-6))	3.048782	-0.066206	1889782.	-0.000965	3.755519	5511412.	3.421836
	(2.99680)	(0.06662)	(2944392)	(0.00083)	(14.8819)	(4470525)	(2.08435)
	[ 1.01735]	[-0.99383]	[ 0.64182]	[-1.15901]	[ 0.25236]	[ 1.23283]	[ 1.64168]
D(PETROLEUMP(-7))	1.438407	-0.042989	-1017124.	0.000521	-0.849019	3665153.	-0.709642
	(2.97894)	(0.06622)	(2926849)	(0.00083)	(14.7932)	(4443889)	(2.07194)
	[ 0.48286]	[-0.64918]	[-0.34752]	[ 0.62910]	[-0.05739]	[ 0.82476]	[-0.34250]
D(PETROLEUMP(-8))	0.063767	-0.090603	-1298432.	0.000773	-3.815467	1693281.	0.791848
	(2.94392)	(0.06544)	(2892437)	(0.00082)	(14.6193)	(4391641)	(2.04758)
	[ 0.02166]	[-1.38448]	[-0.44891]	[ 0.94515]	[-0.26099]	[ 0.38557]	[ 0.38672]
D(STDEBTS(-1))	-2.33E-07	4.42E-10	-0.340961	9.27E-11	-1.30E-06	-0.076491	8.15E-09
	(9.2E-08)	(2.0E-09)	(0.09010)	(2.5E-11)	(4.6E-07)	(0.13680)	(6.4E-08)
	[-2.54022]	[ 0.21671]	[-3.78415]	[ 3.64086]	[-2.85402]	[-0.55913]	[ 0.12773]
D(STDEBTS(-2))	-2.41E-07	3.44E-09	-0.006908	8.26E-11	-1.46E-06	0.251838	2.09E-07
	(9.8E-08)	(2.2E-09)	(0.09624)	(2.7E-11)	(4.9E-07)	(0.14613)	(6.8E-08)
	[-2.46347]	[ 1.58142]	[-0.07178]	[ 3.03735]	[-3.00290]	[ 1.72340]	[ 3.06188]
D(STDEBTS(-3))	4.18E-08	-1.67E-09	0.179478	6.30E-11	-1.07E-06	0.381401	8.00E-08
	(1.0E-07)	(2.3E-09)	(0.10018)	(2.8E-11)	(5.1E-07)	(0.15210)	(7.1E-08)
	[ 0.41012]	[-0.73559]	[ 1.79160]	[ 2.22462]	[-2.11112]	[ 2.50755]	[ 1.12874]
D(STDEBTS(-4))	-5.40E-08	3.01E-10	-0.058196	6.23E-11	3.06E-07	-0.098408	5.92E-08
	(1.0E-07)	(2.3E-09)	(0.10048)	(2.8E-11)	(5.1E-07)	(0.15256)	(7.1E-08)
	[-0.52843]	[ 0.13254]	[-0.57917]	[ 2.19401]	[ 0.60297]	[-0.64503]	[ 0.83216]
D(STDEBTS(-5))	3.13E-08	-3.16E-09	-0.071102	6.49E-11	4.13E-07	-0.305714	-5.08E-08
	(1.0E-07)	(2.3E-09)	(0.10144)	(2.9E-11)	(5.1E-07)	(0.15402)	(7.2E-08)
	[ 0.30354]	[-1.37494]	[-0.70091]	[ 2.26405]	[ 0.80532]	[-1.98489]	[-0.70749]
D(STDEBTS(-6))	-1.00E-07	-5.97E-10	-0.120532	8.66E-11	3.23E-07	-0.344097	-5.27E-09
	(1.1E-07)	(2.3E-09)	(0.10360)	(2.9E-11)	(5.2E-07)	(0.15729)	(7.3E-08)
	[-0.95288]	[-0.25461]	[-1.16347]	[ 2.95542]	[ 0.61772]	[-2.18762]	[-0.07188]
D(STDEBTS(-7))	-7.81E-08	1.46E-10	-0.048722	2.01E-11	-1.83E-07	-0.164531	-6.31E-08
	(9.1E-08)	(2.0E-09)	(0.08965)	(2.5E-11)	(4.5E-07)	(0.13612)	(6.3E-08)
	[-0.85627]	[ 0.07222]	[-0.54346]	[ 0.79190]	[-0.40291]	[-1.20873]	[-0.99374]
D(STDEBTS(-8))	-1.57E-07	-1.55E-09	-0.054822	-4.92E-11	-7.57E-07	-0.125100	3.52E-08
	(8.4E-08)	(1.9E-09)	(0.08221)	(2.3E-11)	(4.2E-07)	(0.12483)	(5.8E-08)
	[-1.87702]	[-0.83188]	[-0.66682]	[-2.11732]	[-1.82239]	[-1.00218]	[ 0.60436]
D(EXRATE(-1))	-2.811008	-2.772873	79905062	-0.491448	1313.893	80263097	41.27888
	(185.638)	(4.12666)	(1.8E+08)	(0.05157)	(921.863)	(2.8E+08)	(129.116)
	[-0.01514]	[-0.67194]	[ 0.43810]	[-9.53018]	[ 1.42526]	[ 0.28983]	[ 0.31970]
D(EXRATE(-2))	93.21819	0.861653	3.90E+08	0.172006	4281.378	5.51E+08	-43.51034
	(206.924)	(4.59985)	(2.0E+08)	(0.05748)	(1027.57)	(3.1E+08)	(143.922)
	[ 0.45049]	[ 0.18732]	[ 1.92056]	[ 2.99242]	[ 4.16651]	[ 1.78530]	[-0.30232]
D(EXRATE(-3))	-1208.155	3.408532	5.54E+08	0.240905	2108.798	9.71E+08	63.35180
	(217.891)	(4.84363)	(2.1E+08)	(0.06053)	(1082.03)	(3.3E+08)	(151.549)
	[-5.54477]	[ 0.70371]	[ 2.58609]	[ 3.98013]	[ 1.94893]	[ 2.98600]	[ 0.41803]
D(EXRATE(-4))	-781.3164	3.148784	6.96E+08	-0.020560	-758.2999	1.55E+09	-8.299680
	(246.241)	(5.47384)	(2.4E+08)	(0.06840)	(1222.81)	(3.7E+08)	(171.267)
	[-3.17297]	[ 0.57524]	[ 2.87791]	[-0.30057]	[-0.62013]	[ 4.21448]	[-0.04846]
D(EXRATE(-5))	420.1037	-2.188715	-1.26E+09	-0.008675	-2734.381	-7.48E+08	-2.383399
	(242.937)	(5.40039)	(2.4E+08)	(0.06748)	(1206.41)	(3.6E+08)	(168.969)
	[ 1.72927]	[-0.40529]	[-5.28345]	[-0.12855]	[-2.26655]	[-2.06348]	[-0.01411]
D(EXRATE(-6))	393.2689	-0.454682	-7.94E+08	0.265610	-521.5082	-9.26E+08	314.3096
	(240.869)	(5.35441)	(2.4E+08)	(0.06691)	(1196.13)	(3.6E+08)	(167.531)
	[ 1.63271]	[-0.08492]	[-3.35323]	[ 3.96967]	[-0.43599]	[-2.57819]	[ 1.87613]
D(EXRATE(-7))	600.3543	7.976416	6.62E+08	0.211517	-848.2996	7.83E+08	240.4917
	(240.335)	(5.34255)	(2.4E+08)	(0.06676)	(1193.48)	(3.6E+08)	(167.160)
	[ 2.49799]	[ 1.49300]	[ 2.80483]	[ 3.16825]	[-0.71078]	[ 2.18278]	[ 1.43870]
D(EXRATE(-8))	588.6773	0.672860	7.46E+08	0.017354	811.6029	1.22E+09	-174.1170
	(235.575)	(5.23674)	(2.3E+08)	(0.06544)	(1169.85)	(3.5E+08)	(163.849)
	[ 2.49890]	[ 0.12849]	[ 3.22334]	[ 0.26519]	[ 0.69377]	[ 3.47689]	[-1.06267]
D(M2(-1))	0.058149	-0.000443	-57250.22	-4.44E-05	0.009591	-93227.38	0.014736
	(0.02176)	(0.00048)	(21376.2)	(6.0E-06)	(0.10804)	(32455.9)	(0.01513)
	[ 2.67272]	[-0.91511]	[-2.67822]	[-7.35184]	[ 0.08877]	[-2.87243]	[ 0.97380]
D(M2(-2))	0.068845	-0.000742	-39212.94	-2.51E-05	-0.769436	-97876.62	0.003670
	(0.02319)	(0.00052)	(22787.0)	(6.4E-06)	(0.11517)	(34597.9)	(0.01613)
	[ 2.96842]	[-1.43912]	[-1.72085]	[-3.89476]	[-6.68073]	[-2.82898]	[ 0.22753]
D(M2(-3))	0.056133	-0.000651	-75715.82	-3.35E-05	0.400480	-122348.9	0.018433
	(0.02793)	(0.00062)	(27437.2)	(7.8E-06)	(0.13868)	(41658.4)	(0.01942)
	[ 2.01011]	[-1.04909]	[-2.75960]	[-4.31918]	[ 2.88788]	[-2.93695]	[ 0.94905]
D(M2(-4))	0.064225	-0.001250	-87037.80	-2.75E-05	-0.408591	-175399.8	0.001125
	(0.03217)	(0.00072)	(31605.1)	(8.9E-06)	(0.15974)	(47986.5)	(0.02237)
	[ 1.99657]	[-1.74740]	[-2.75392]	[-3.08207]	[-2.55782]	[-3.65519]	[ 0.05027]
D(M2(-5))	0.090185	-0.001136	-153484.2	-5.13E-05	0.054543	-229598.0	0.017564
	(0.03696)	(0.00082)	(36318.0)	(1.0E-05)	(0.18356)	(55142.4)	(0.02571)
	[ 2.43976]	[-1.38248]	[-4.22611]	[-4.99725]	[ 0.29713]	[-4.16373]	[ 0.68317]
D(M2(-6))	0.113449	-0.000688	-60294.97	-2.97E-05	-1.274839	-177245.7	-0.012463
	(0.05385)	(0.00120)	(52903.8)	(1.5E-05)	(0.26739)	(80324.8)	(0.03745)
	[ 2.10694]	[-0.57478]	[-1.13971]	[-1.98700]	[-4.76768]	[-2.20661]	[-0.33279]
D(M2(-7))	0.019836	0.000209	-6404.737	-6.80E-05	0.345127	50409.06	0.036011
	(0.05055)	(0.00112)	(49666.8)	(1.4E-05)	(0.25103)	(75410.0)	(0.03516)
	[ 0.39240]	[ 0.18562]	[-0.12895]	[-4.84473]	[ 1.37484]	[ 0.66847]	[ 1.02421]
D(M2(-8))	-0.024762	-0.000504	53677.54	-6.44E-05	-0.921609	41474.31	-0.005815
	(0.04544)	(0.00101)	(44643.8)	(1.3E-05)	(0.22564)	(67783.5)	(0.03160)
	[-0.54495]	[-0.49890]	[ 1.20235]	[-5.10196]	[-4.08436]	[ 0.61186]	[-0.18400]
D(FDI(-1))	-6.27E-08	3.80E-10	-0.046140	1.12E-12	3.60E-07	-0.316074	-2.85E-08
	(6.2E-08)	(1.4E-09)	(0.06056)	(1.7E-11)	(3.1E-07)	(0.09196)	(4.3E-08)
	[-1.01692]	[ 0.27725]	[-0.76184]	[ 0.06548]	[ 1.17662]	[-3.43726]	[-0.66435]
D(FDI(-2))	-8.44E-08	-9.98E-10	-0.067348	-5.44E-12	2.19E-07	-0.178124	-9.77E-08
	(6.3E-08)	(1.4E-09)	(0.06183)	(1.7E-11)	(3.1E-07)	(0.09387)	(4.4E-08)
	[-1.34175]	[-0.71350]	[-1.08927]	[-0.31093]	[ 0.70116]	[-1.89746]	[-2.23193]
D(FDI(-3))	-2.93E-07	1.44E-09	-0.074194	1.08E-11	2.13E-07	-0.092947	-2.71E-09
	(6.3E-08)	(1.4E-09)	(0.06199)	(1.8E-11)	(3.1E-07)	(0.09413)	(4.4E-08)
	[-4.64218]	[ 1.02508]	[-1.19681]	[ 0.61815]	[ 0.67821]	[-0.98748]	[-0.06178]
D(FDI(-4))	-2.56E-07	-5.47E-10	0.073559	5.28E-11	-4.18E-07	0.157817	3.40E-08
	(6.7E-08)	(1.5E-09)	(0.06569)	(1.9E-11)	(3.3E-07)	(0.09973)	(4.7E-08)
	[-3.83614]	[-0.36809]	[ 1.11983]	[ 2.84475]	[-1.25878]	[ 1.58237]	[ 0.73150]
D(FDI(-5))	-1.82E-07	1.70E-09	0.104080	1.05E-10	-3.94E-07	0.280771	2.12E-08
	(6.9E-08)	(1.5E-09)	(0.06747)	(1.9E-11)	(3.4E-07)	(0.10244)	(4.8E-08)
	[-2.65214]	[ 1.11551]	[ 1.54263]	[ 5.47886]	[-1.15487]	[ 2.74086]	[ 0.44469]
D(FDI(-6))	-8.49E-08	4.43E-10	0.089501	8.95E-11	-2.31E-07	0.234547	1.13E-08
	(7.1E-08)	(1.6E-09)	(0.06932)	(2.0E-11)	(3.5E-07)	(0.10525)	(4.9E-08)
	[-1.20355]	[ 0.28246]	[ 1.29107]	[ 4.56738]	[-0.65846]	[ 2.22839]	[ 0.23032]
D(FDI(-7))	-8.23E-08	-2.19E-10	0.007135	1.06E-10	-5.52E-07	0.062418	7.34E-08
	(6.5E-08)	(1.4E-09)	(0.06392)	(1.8E-11)	(3.2E-07)	(0.09705)	(4.5E-08)
	[-1.26453]	[-0.15138]	[ 0.11162]	[ 5.89142]	[-1.71010]	[ 0.64314]	[ 1.62245]
D(FDI(-8))	2.95E-08	1.35E-09	-0.027145	1.57E-10	-4.81E-08	0.032107	2.45E-08
	(6.1E-08)	(1.3E-09)	(0.05958)	(1.7E-11)	(3.0E-07)	(0.09046)	(4.2E-08)
	[ 0.48698]	[ 1.00207]	[-0.45562]	[ 9.29118]	[-0.15978]	[ 0.35494]	[ 0.58161]
D(COCOAP(-1))	-0.029110	0.001236	-96866.71	-4.87E-06	-0.007408	-106858.3	0.065403
	(0.09270)	(0.00206)	(91076.7)	(2.6E-05)	(0.46033)	(138283.)	(0.06447)
	[-0.31403]	[ 0.59991]	[-1.06357]	[-0.18915]	[-0.01609]	[-0.77275]	[ 1.01441]
D(COCOAP(-2))	0.028935	0.004015	21211.19	5.70E-06	-0.728342	-126078.9	-0.036148
	(0.09115)	(0.00203)	(89557.7)	(2.5E-05)	(0.45265)	(135977.)	(0.06340)
	[ 0.31743]	[ 1.98168]	[ 0.23684]	[ 0.22515]	[-1.60906]	[-0.92721]	[-0.57017]
D(COCOAP(-3))	-0.044734	-0.000844	-9854.080	2.21E-05	0.145310	-26634.72	0.006988
	(0.09196)	(0.00204)	(90347.2)	(2.6E-05)	(0.45664)	(137176.)	(0.06396)
	[-0.48648]	[-0.41312]	[-0.10907]	[ 0.86656]	[ 0.31821]	[-0.19416]	[ 0.10926]
D(COCOAP(-4))	0.104775	0.000632	-14689.21	-4.72E-06	-0.277008	-622.0302	0.008293
	(0.08918)	(0.00198)	(87622.5)	(2.5E-05)	(0.44287)	(133039.)	(0.06203)
	[ 1.17484]	[ 0.31895]	[-0.16764]	[-0.19034]	[-0.62548]	[-0.00468]	[ 0.13370]
D(COCOAP(-5))	-0.113259	0.000743	-40334.98	-3.39E-05	0.863673	-51758.91	-0.057151
	(0.08823)	(0.00196)	(86687.4)	(2.5E-05)	(0.43814)	(131619.)	(0.06137)
	[-1.28367]	[ 0.37859]	[-0.46529]	[-1.38227]	[ 1.97121]	[-0.39325]	[-0.93130]
D(COCOAP(-6))	-0.114153	-0.003723	31012.82	3.22E-05	-0.789703	81798.85	-0.030308
	(0.08874)	(0.00197)	(87187.2)	(2.5E-05)	(0.44067)	(132378.)	(0.06172)
	[-1.28639]	[-1.88720]	[ 0.35570]	[ 1.30765]	[-1.79205]	[ 0.61792]	[-0.49105]
D(COCOAP(-7))	0.046109	-0.000853	53512.35	1.52E-05	0.106539	100266.5	0.128226
	(0.08849)	(0.00197)	(86942.8)	(2.5E-05)	(0.43944)	(132007.)	(0.06155)
	[ 0.52106]	[-0.43387]	[ 0.61549]	[ 0.61692]	[ 0.24245]	[ 0.75956]	[ 2.08337]
D(COCOAP(-8))	-0.092239	-0.004356	132197.5	4.84E-05	-0.332617	235911.9	-0.139993
	(0.08784)	(0.00195)	(86306.6)	(2.4E-05)	(0.43622)	(131041.)	(0.06110)
	[-1.05004]	[-2.23059]	[ 1.53172]	[ 1.98397]	[-0.76250]	[ 1.80029]	[-2.29133]
C	4.300793	0.692892	88433757	0.027118	591.7129	1.14E+08	-20.21442
	(23.4991)	(0.52237)	(2.3E+07)	(0.00653)	(116.694)	(3.5E+07)	(16.3442)
	[ 0.18302]	[ 1.32643]	[ 3.83027]	[ 4.15434]	[ 5.07062]	[ 3.25474]	[-1.23679]
R-squared	0.837320	0.862851	0.661764	0.581940	0.715773	0.471400	0.536804
Adj. R-squared	0.674812	0.722754	0.585991	0.494851	0.428576	0.355286	0.433699
Sum sq. resids	5169122.	165101.8	0.614172	1.97E+08	0.148745	1.77E+19	7.77E+18
S.E. equation	301.1417	53.81936	0.048233	864.2645	0.051084	2.59E+08	1.72E+08
F-statistic	5.114508	6.168867	8.857936	6.438541	2.487099	4.059416	5.251769
Log likelihood	-785.4650	-585.7191	553.5018	-2609.829	221.6316	-6682.704	-6549.938
Akaike AIC	14.55974	11.11585	-3.061931	16.52526	-2.803992	41.74429	40.92222
Schwarz SC	15.96027	12.51638	-2.371895	17.21529	-1.403460	42.43433	41.61226
Mean dependent	262.8103	2.236293	0.013335	144.3161	-0.001379	36121893	28325139
S.D. dependent	528.0847	102.2130	0.074961	1216.009	0.067578	3.22E+08	2.28E+08
Determinant resid covariance (dof adj.)		8.51E+45					
Determinant resid covariance		2.07E+45					
Log likelihood		-20059.99					
Akaike information criterion		126.8668					
Schwarz criterion		131.8842					

## Appendix B Supplementary data

dataa p2 dataa p2
